# CRABP2 Promotes the Proliferation of Dermal Papilla Cells via the Wnt/β-Catenin Pathway

**DOI:** 10.3390/ani13122033

**Published:** 2023-06-19

**Authors:** Mingliang He, Xiaoyang Lv, Xiukai Cao, Zehu Yuan, Kai Quan, Tesfaye Getachew, Joram M. Mwacharo, Aynalem Haile, Yutao Li, Shanhe Wang, Wei Sun

**Affiliations:** 1 College of Animal Science and Technology, Yangzhou University, Yangzhou 225009, China; 2 Joint International Research Laboratory of Agriculture and Agri-Product Safety, Ministry of Education of China, Yangzhou University, Yangzhou 225009, China; 3 International Joint Research Laboratory in Universities of Jiangsu Province of China for Domestic Animal Germplasm Resources and Genetic Improvement, Yangzhou University, Yangzhou 225009, China; 4 College of Animal Science and Technology, Henan University of Animal Husbandry and Economy, Zhengzhou 450046, China; 5 International Centre for Agricultural Research in the Dry Areas, Addis Ababa 999047, Ethiopia; 6 CSIRO Agriculture and Food, 306 Carmody Rd., St. Lucia, QLD 4067, Australia

**Keywords:** CRABP2, DPCs, proliferation, Wnt/β-catenin

## Abstract

**Simple Summary:**

Dermal papilla cells are a vital cell type in the hair follicles. It has been reported that the number of dermal papilla cells could affect the growth and development of hair follicles. Our previous study found that the CRABP2 gene is highly expressed in dermal papilla cells, but its function in dermal papilla cells is still unclear. In this study, we detected the role of the CRABP2 gene in the proliferation of dermal papilla cells and its effect on the Wnt/β-Catenin pathway. Our findings indicate that CRABP2 could promote the proliferation of dermal papilla cells by activating the Wnt/β-Catenin pathway.

**Abstract:**

In our previous study of Hu sheep hair follicles, we found that CRABP2 was highly expressed in DPCs, which suggested that CRABP2 may influence the number of DPCs. In the present study, we aimed to understand the effect of CRABP2 in Hu sheep dermal papilla cells (DPCs). First, we explored the influence of CRABP2 on the ability of Hu sheep DPCs’ proliferation. Based on the results obtained from some experiments, such as CCK-8, EDU, qPCR, and Western blot experiment, we found that the overexpression of CRABP2 facilitated the proliferation of DPCs compared to the negative control group. Then, we also detected the effect of CRABP2 on the Wnt/β-catenin pathway based on the important function of the Wnt/β-catenin pathway in hair follicles. The results showed that CRABP2 could activate the Wnt/β-catenin pathway in DPCs, and it rescues the proliferation of DPCs when the Wnt/β-catenin pathway was inhibited. In summary, our findings indicate that CRABP2 is a vital functional gene in the proliferation of Hu sheep DPCs. Our study will be of great use for revealing the roles of CRABP2 in the hair follicles of Hu sheep.

## 1. Introduction

As the only source of wool products, wool production has a significant impact on the world textile industry [1,2]. As everyone knows, wool grows from hair follicles, so the growth of hair follicles inevitably affects the production of wool. The hair follicle is a structure composed of hair follicle epithelial cells and hair follicle mesenchymal cells. The hair follicle epithelium includes the hair shaft, the inner root sheath, the accompanying layer, the outer root sheath, and the hair matrix. The follicular stroma includes the dermal papilla and connective tissue sheath. All these cells participate in the regulation process of hair follicle growth, differentiation, and periodic growth through mutual regulation [3,4]. The dermal papilla is in the central region of the hair bulb, and it can activate the hair matrix cells to maintain and initiate the hair follicle growth cycle [5]. Dermal papilla cells are thought to regulate HF growth through a paracrine mechanism, in which exosomes may play a key role [6]. By extracting exosomes from 2D and 3D cultured dermal papilla cells, Kwack et al. found that the extracted exosomes could promote the proliferation of dermal papilla cells and outer root sheath cells, induce the telogen phase of mouse hair to the growth phase, increase hair shaft elongation, and enhance hair follicle regeneration in human hair follicles, which suggests that dermal papilla cells can promote hair growth through their exosomes regulating the activity of other cells in the hair follicle [7]. Furthermore, it has been reported that the presence of dermal papilla contributes to the formation of intact hair follicles and promotes hair growth [8]. Therefore, it is helpful to understand the mechanism of hair follicle growth by studying the proliferation regulation mechanism of dermal papilla cells.

Cellular retinoic acid binding protein 2 (CRABP2) is a member of the specific carrier protein family of vitamin A [9]. CRABP2 is a low-molecular-mass (15 kDa) protein with a high affinity for RA and is involved in several biological processes, including differentiation, proliferation, and apoptosis, by binding RA and its related molecules to stimulate the RA pathway [10,11]. CRABP2 was proven key to the normal development and maintenance of motor neurons in the spinal cord of mice. It is expressed in the central nervous system during zebrafish embryonic development [12,13]. In addition, it has been reported that CRABP2 is also expressed in epithelial and mesenchymal cells within hair follicles and is involved in β-catenin pathways and interactions between epithelial and mesenchymal cells; all of this suggests that CRABP2 may play a potential role in hair follicles [14,15]. Interestingly, we found a high expression of CRABP2 in hair follicles’ dermal papilla cells in a previous study by our team [16]. That finding convinced us that CRABP2 may be important in forming Hu sheep wool.

The Wnt signal pathway mainly includes three branches, namely, the classical Wnt signal pathway and the non-classical signal pathway, where the classical Wnt signal pathway is the Wnt/β-Catenin signal pathway and non-classical signaling pathways include the Wnt/PCP pathway and the Wnt/Ca^2+^ pathway [17]. Research shows that the classic Wnt/β-catenin pathway plays an important role in the induction of hair follicle formation, initiating hair follicle morphogenesis and maintaining and inducing hair follicle dermal papilla cell regeneration and hair stem growth [18,19]. Meanwhile, some factors have reportedly been involved in the proliferation of hair papilla cells and hair follicle epithelial cells via the Wnt/β-catenin pathway. Wnt10b induces the transition of DPCs to the G1/S phase and upregulates β-catenin protein, promoting the proliferation of DPCs in Rex rabbits [20]. MiR-130b-3p can regulate the proliferation of fetal skin hair follicle epithelial cells and dermal fibroblasts in Inner Mongolia cashmere goats by targeting WNT10A [21]. In addition, it has also been reported that CRABP2 is relevant to β-catenin pathways [15]. All of these suggest that we need to investigate the effect of CRABP2 on the proliferation of DPCs and the Wnt/β-catenin pathway in DPCs.

Our research aimed to understand the effect of CRABP2 on the proliferation of Hu sheep dermal papilla cells. The results of this study are helpful to understand the mechanism of sheep hair follicle growth.

## 2. Materials and Methods

### 2.1. Ethics Statement

In this study, all animal experimental protocols were designed in strict accordance with the “Jiangsu Province laboratory animal management measures”, and the Animal Ethics Committee of Yangzhou University approved all protocols (Approval number: No. 202103279).

### 2.2. Animals, Cell Isolation, and Culture

The Hu sheep used for cell isolation were provided by Suzhou Sheep Farm (Suzhou, Jiangsu, China). Isolation of Hu sheep dermal papilla cells: we used ophthalmic scissors to cut the skin tissue, and then individual hair follicles were isolated from small pieces of skin tissue. Next, we used tweezers to acquire the swollen end of the hair follicle and extract the dermal papilla (DP) from it. Finally, we seeded the swollen hair follicles’ ends into a 12-well plate until dermal papilla cells grew.

Culture process of Hu sheep dermal papilla cells: we inoculated well-growing dermal papilla cells into cell culture plates and added specially formulated culture medium to ensure their growth. The medium for culture Hu sheep dermal papilla cells was DMEM-F12 (Sigma-Aldrich, St. Louis, MO, USA) containing 10% fetal bovine serum (Gibco, Grand Island, NY, USA) and 1% penicillin–streptomycin–amphotericin (Solarbio, Beijing, China). All Hu sheep dermal papilla cells were cultured in a cell incubator (Thermo, Waltham, MA, USA) with 5% CO_2_ at 37 °C.

### 2.3. RNA Extraction, cDNA Synthesis, and qRT-PCR

Total RNA was extracted from dermal papilla cells using Trizol (Takara, Dalian, China). We used a spectrophotometer (Thermo, Waltham, MA, USA) to detect the quality and concentration of RNA. After quality testing and concentration determination, the RNA was stored in a freezer at −80 degrees Celsius for subsequent experiments. The cDNA synthesis of genes was performed using a one-step reverse transcription Kit (Tiagen, Beijing, China). The expression levels of related genes in tissues and cells were detected using a 2× TSINGKE ^®^ Master qPCR mix (Tsingke, Nanjing, China). Sheep β-actin was used as reference genes, and three repeated tests were performed for each sample. 2^−ΔΔCT^ method was used to calculate relative expression [22].

### 2.4. Primers for qRT-PCR

This study used Premier Primer 5.0 software (Premier Biosoft International, Palo Alto, CA, USA) to design primers of related genes. These primers were synthesized using Tsingke (Nanjing, China), and these primers are shown in Table 1.

### 2.5. Plasmid Construction

For the construction of the overexpression vector of CRABP2, we first amplified a full-length coding domain sequence (CDS) of the CRABP2 gene using PrimeSTAR^®^ Max DNA Polymerase (Takara, Dalian, China), and we separated and purified the PCR product. Then, we cloned the amplified CDS fragment of the CRABP2 gene into the pcDNA 3.1+ reporting vector. The restriction sites were HindⅢ and EcoRI. We sequenced the recombinant plasmid and ensured that the fragment was inserted successfully. Primers are shown in Table 2.

### 2.6. Cell Transfection

In this study, the jetPRIME Transfection Reagent (polyplus, illkirch, France) was used for cell transfection. All cell transfection procedures were in accordance with the manufacturer’s protocol.

### 2.7. CCK-8 Assay

For the CCK-8 assay, we planted DPCs using a 96-well cell culture plate. After the DPCs reached 30% density, cells were transfected. According to the instruction manual of CCK-8 Kit (Vazyme, Nanjing, China), cell viability was detected at 12 h (after DPC transfected), 24 h, 36 h, and 48 h. The multi-mode micropore detection system (EnSpire, PerkinElmer, Waltham, MA, USA) was used to detect the absorbance at 450 nm.

### 2.8. EdU Assay

For the EdU assay, we planted DPCs using a 24-well cell culture dish. After the DPCDPC reached 50% density, cells were transfected. After 2 days, we used 4% paraformaldehyde (Solarbio, Beijing, China) to fix DPCs for 30 min. Then, we used Triton X-100 to make cell membrane permeabilization. Finally, we treated DPCs using an EdU Apollo In Vitro Imaging Kit (RiboBio, Guangzhou, China). After the DPCs were dyed, we used a fluorescence-inverted microscope (DMi8, Leica, Germany) to acquire images of stained cells. Three regions were randomly selected for evaluating the number of stained cells, and Image Pro Plus 6.0 software (Media Cybernetics, Rockville, MD, USA) was used for data analysis.

### 2.9. Immunofluorescence Assay

For the immunofluorescence assay, we planted DPCs using a 24-well cell culture dish. After the DPCs reached 50% density, cells were transfected according to the cell transfection procedure. The cells used for marker gene expression identification were not transfected. When DPCs reached a suitable density, we washed them using 1× PBS (Solarbio, Beijing, China) to ensure a good cell fixation effect. Then, 4% paraformaldehyde (Solarbio, Beijing, China) was used to fix the cell after DPCs were washed, and the cells were permeated using 0.5% Triton X-100 (Solarbio, Beijing, China) after DPCs were fixed. After the cell permeability was complete, we cleaned the cells with 1× PBS and sealed them with 5% BSA (Solarbio, Beijing, China) in a 37 °C environment. Finally, we used the primary antibody to incubate the cells in a dark environment at 4 °C. After 12 h, we washed the cells using 1× PBST (Solarbio, Beijing, China), and then the corresponding secondary antibody was used to incubate the cells in a dark environment at 37 °C. For the nuclei of DPC, DAPI (Beyotime, Shanghai, China) was used for staining. To obtain cell staining status, a fluorescence-inverted microscope (Nikon, Tokyo, Japan) was used to observe and photograph them. The primary antibodies and their dilution ratios are as follows: anti-ACTA1 antibody (Sangon Biotech, Shanghai, China, 1:400) and anti-VIM antibody (Santa Cruz Biotech, Santa Cruz, The Republic Bolivia, 1:400). The secondary antibodies and their dilution ratios were Goat Anti-Rabbit IgG H&L (Alexa Fluor^®^ 594), (Abcam, Cambridge, UK, 1:400)

### 2.10. TOP/FOP-Flash Wnt Report Assays

The β-catenin/TCF transcription activity in DPCs was estimated using TOP/FOP-flash plasmid (Beyotime, Shanghai, China). After the DPCs reached 50% density, we co-transfected pcDNA 3.1-CRABP2/pcDNA 3.1, TOP/FOP-flash plasmid, and the Renilla luciferase reporter plasmid (pRL-TK) into DPCs. After 48 h of transfection, we used a dual-luciferase detection kit (Vazyme, Nanjing, China) to process the cells. Then, we detected the Luciferase activity using a multi-mode micropore detection system (EnSpire, Perkin Elmer, Waltham, MA, USA).

### 2.11. Western Blot

After the DPCs reached 50% density, cells were transfected. We used RIP cell lysates (Beyotime, Shanghai, China) to lyse DPCs after they were transfected 48 h later. Then, we collected cell lysate using a 1.5 mL enzyme-free centrifuge tube. After the collection of cell lysates was completed, we used a high-speed low-temperature centrifuge to centrifuge the cell lysates at a speed of 12,000 rpm and collected the supernatant again using a new 1.5 mL enzyme-free centrifuge tube. Proteins were contained in the supernatant of cell lysates, so we used the BCA Protein Quantification Kit (Vazyme, Nanjing, China) to determine the concentration of proteins in the supernatant. Then, we used an SDS-PAGE Loading Buffer (New Cell & Molecular Biotech, Suzhou, China) to denature the protein sample for electrophoresis. After SDS-PAGE polyacrylamide gel electrophoresis, we transferred the obtained target protein to PVDF membranes (BIO-RAD, Hercules, CA, USA). After the transfer process was completed, we used 5% skimmed milk powder to incubate the PVDF membrane containing the target protein at room temperature for 1 h (Beyotime, Shanghai, China). Finally, we used the primary antibody to incubate the sealed PVDF membrane overnight at 4 °C. After 12–16 h, we washed the PVDF membrane incubated with the primary antibody using 1× TBST (Solarbio, Beijing, China). Then, we continued to incubate the PVDF membrane with the secondary antibody. Similarly, we washed the PVDF membrane with PBST after the secondary antibody incubation. We used the enhanced chemiluminescence method (ECL) method to imprint and display the PVDF membrane after treatment. To obtain the relative expression of proteins, the ChemDocTMTouch imaging system (Bio-Rad, Hercules, CA, USA) was used to photograph and analyze PVDF membranes. The primary antibodies and their dilution ratios are as follows: PCNA (Affinity Biosciences, Cincinnati, OH, USA, 1:1000), β-actin (Affinity Biosciences, Cincinnati, OH, USA, 1:5000), and β-catenin (Beyotime, Shanghai, China, 1:1000). The secondary antibodies and their dilution ratios are as follows: Goat Anti-Rabbit IgG H&L (HRP) (Abcam, Cambridge, UK, 1:5000) and Rabbit Anti-Mouse IgG H&L (HRP) (Abcam, Cambridge, UK, 1:5000).

### 2.12. Statistical Analysis

Statistical analysis was performed using SPSS 25.0 software (SPSS Inc., Chicago, IL, USA). Analysis of two-group comparison using the unpaired Student’s *t*-test was used. Only when *p* < 0.05 (*), *p* < 0.01 (**), or *p* < 0.001 (***) were the data considered statistically significant. In this study, assays were repeated three times in each experiment. All data provided in this study are presented as means ± SEM (standard error of the mean).

## 3. Results

### 3.1. Marker Gene Identification in Hu Sheep DPCs

**Figure 1:**
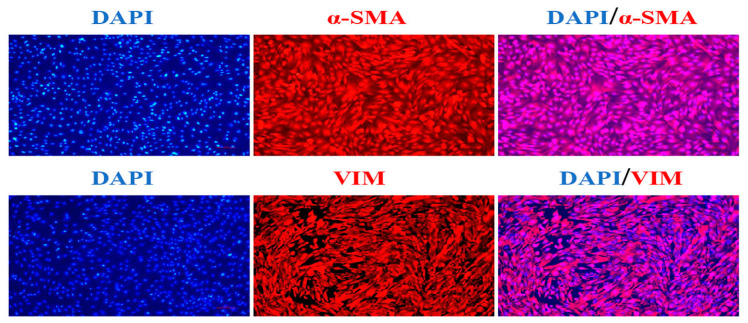
Marker gene identification in Hu sheep DPCs (the scale is 50 μm).

To ensure that the isolated cells were dermal papilla cells, two genes (a-SMA and Vimentin) were selected to identify Hu sheep DPCs. The immunofluorescence staining results showed that the extracted cells were of high purity and could be used for subsequent experiments (Figure 1).

### 3.2. Overexpression of CRABP2 Promotes DPC Proliferation

**Figure 2:**
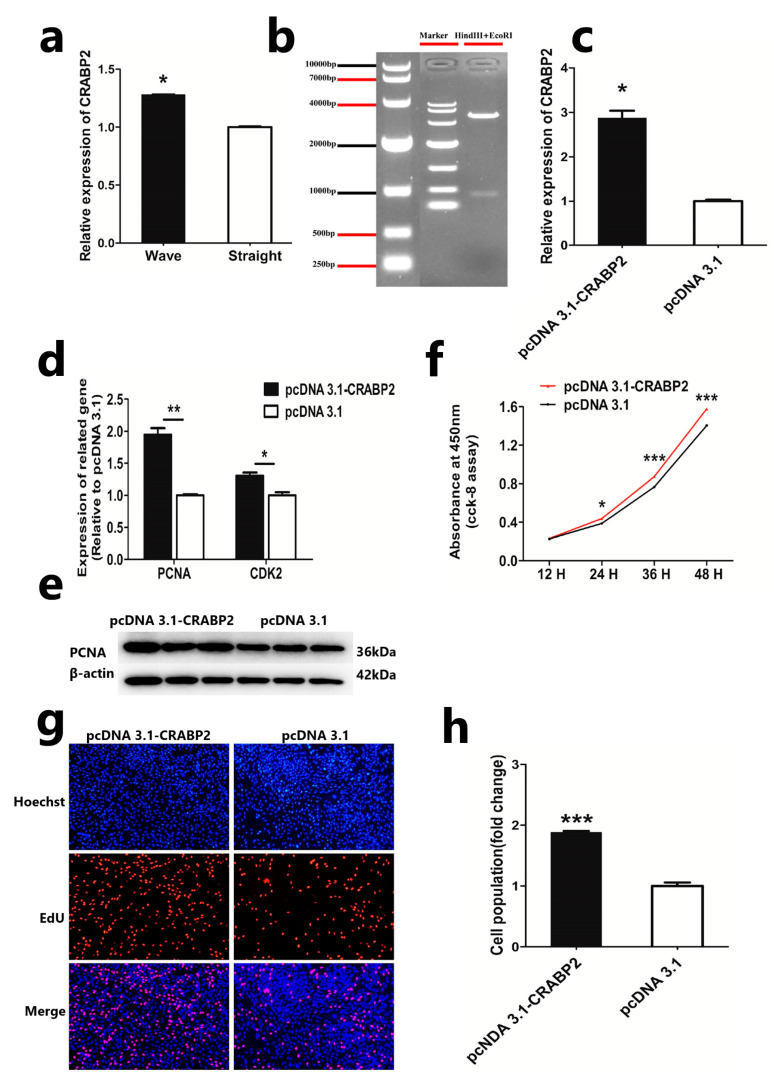
Overexpression of CRABP2 promotes DPC proliferation. (**a**) Expression of CRABP2 in the dermal papilla cells from different hair phenotypes’ skin in Hu sheep. (**b**) Gel electrophoresis of overexpression vector plasmid digested by double enzyme digestion. (**c**) The mRNA expression of CRABP2 after overexpression of it in DPCs. (**d**) The mRNA expression level of PCNA and CDK2 after overexpression of CRABP2 in sheep DPCs. (**e**) The protein expression level of PCNA after overexpression of CRABP2 in sheep DPCs. (**f**) CCK-8 assay after overexpression of CRABP2 in sheep DPCs. (**g**,**h**) EdU assay after overexpression of CRABP2 in sheep DPCs; the scale is 100 μm. Data are expressed as means ± SEM (standard error of the mean) (*n* = 3). The unpaired Student’s *t*-test was used for Statistical significance. (* *p* < 0.05; ** *p* < 0.01; *** *p* < 0.001).

In our previous study, we found that CRABP2 is highly expressed in hair follicle papilla cells, and it is also considered to be a marker gene for DPCs in Hu sheep [16,23]. We also detected the expression of CRABP2 in the dermal papilla cells from different hair skin phenotypes in Hu sheep. The results showed that CRABP2 was highly expressed in the DPCs from curly-hair phenotypes’ skin (Figure 2a). All of these suggest that CRABP2 may be an important factor in the formation of Hu sheep’s different hair phenotypes. Therefore, we constructed the overexpression plasmid of CRABP2 to research its function in Hu sheep DPCs. Double restriction enzyme digestion and vector sequencing showed that the construction of the CRABP2 overexpression plasmid was successful (Figure 2b). Then, we transfected it to DPCs and detected the mRNA expression of CRABP2. QRT-PCR showed that the expression level of CRABP2 was significantly increased (Figure 2c). These results showed that the overexpression vector of CRABP2 was successfully constructed. To further understand the effect of CRABP2, we detected the effect of CRABP2 using CCK-8, EDU, qPCR, and Western blot. The result of qRT-PCR showed that overexpression of CRABP2 could significantly increase the mRNA level of PCNA and CDK2 (Figure 2d). Also, the protein level of PCNA increased after overexpression of CRABP2 in DPCs (Figure 2e). CCK8 and EDU assays showed that overexpression of CRABP2 could facilitate cell vitality and cell proliferation, respectively (Figure 2f–h). These results preliminarily showed that the overexpression of CRABP2 was beneficial to the proliferation of DPCs.

### 3.3. CRABP2 Regulates the Proliferation of DPCs via the Wnt/β-Catenin Pathway

**Figure 3:**
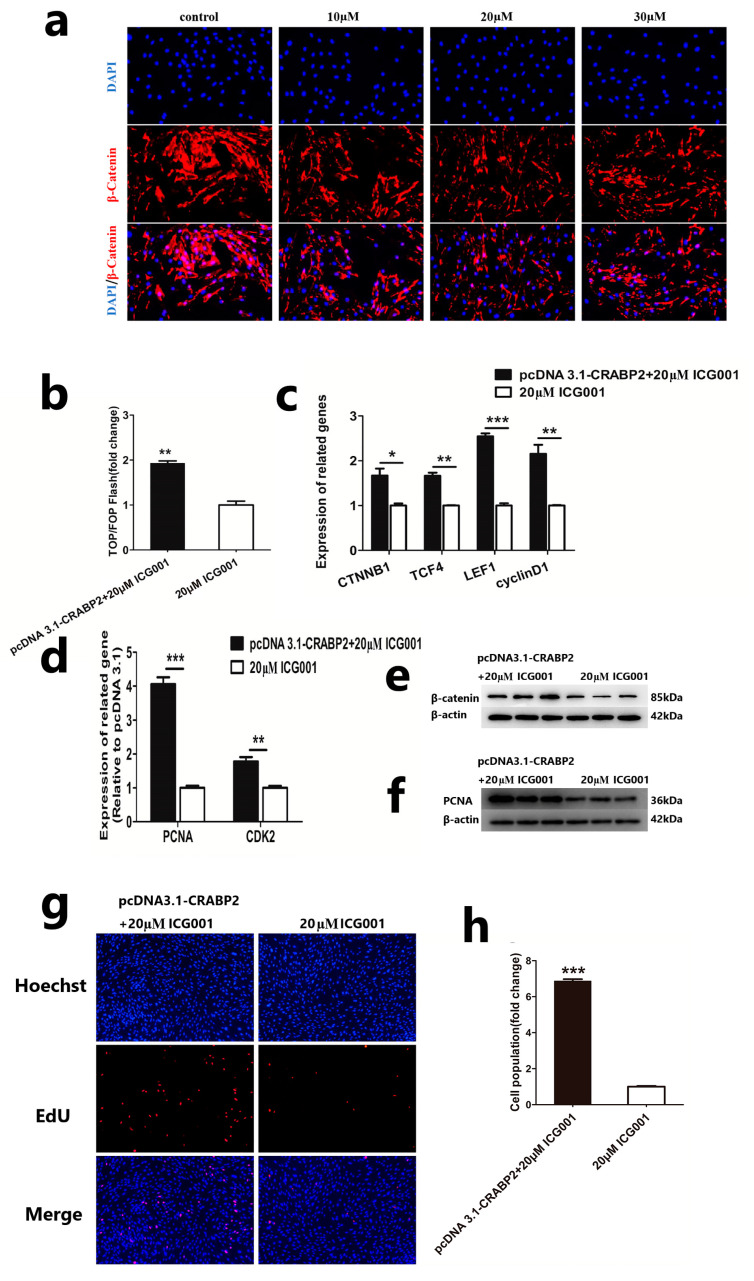
CRABP2 regulates the proliferation of DPCs via the Wnt/β-catenin pathway. (**a**) Immunofluorescence staining of β-catenin after adding 10 μM, 20 μM, or 30 μM ICG001 to DPCs; the scale is 50 μm. (**b**) TOP/FOP-flash assays of wnt/β-catenin activation after DPC was handled. (**c**,**d**) qRT-PCR assay of Wnt/β-catenin-pathway-related genes and cell-proliferation-related genes after DPCs were handled. (**e**,**f**) The protein expression level of β-catenin and PCNA after DPCs were handled. (**g**,**h**) EdU assay after DPCs were handled; the scale is 100 μm. Data are expressed as means ± SEM (standard error of the mean) (*n* = 3). The unpaired Student’s *t*-test was used for Statistical significance. (* *p* < 0.05; ** *p* < 0.01; *** *p* < 0.001).

Due to the positive role of the Wnt/β-catenin pathway in cell proliferation, we investigated whether CRABP2 affects the increase in DPCs through the Wnt/β-catenin pathway. First, we inhibited the activation of the Wnt/β-catenin pathway by adding a Wnt/β-catenin pathway inhibitor (ICG001, Beyotime, Shanghai, China) in DPCs. We found that 20 μM ICG001 could decrease the expression of β-catenin in DPCs greatly compared to the control group (Figure 3a). Then, we transfected pcDNA 3.1-CRABP2 to DPCs, adding 20 μM ICG001 and detected relevant indicators. The control group was no pcDNA 3.1-CRABP2 transfected. We found that overexpression of CRABP2 could significantly rescue the activation of Wnt/β-catenin after DPC adding the Wnt/β-catenin pathway inhibitor (Figure 3b). The results of qRT-PCR showed that overexpressing CRABP2 could increase the mRNA expression of Wnt/β-catenin-related genes and cell-proliferation-related genes compared to the control group (Figure 3c,d). Meanwhile, the protein expression of β-catenin and PCNA was increased after transfected pcDNA 3.1-CRABP2 to DPCs adding 20 μM ICG001(Figure 3e,f). EdU assay showed that overexpression of CRABP2 could significantly facilitate the proliferation of DPCs after inhibiting the Wnt/β-catenin pathway (Figure 3g,h).

### 3.4. CRABP2 Activates the Wnt/β-Catenin Pathway 

**Figure 4:**
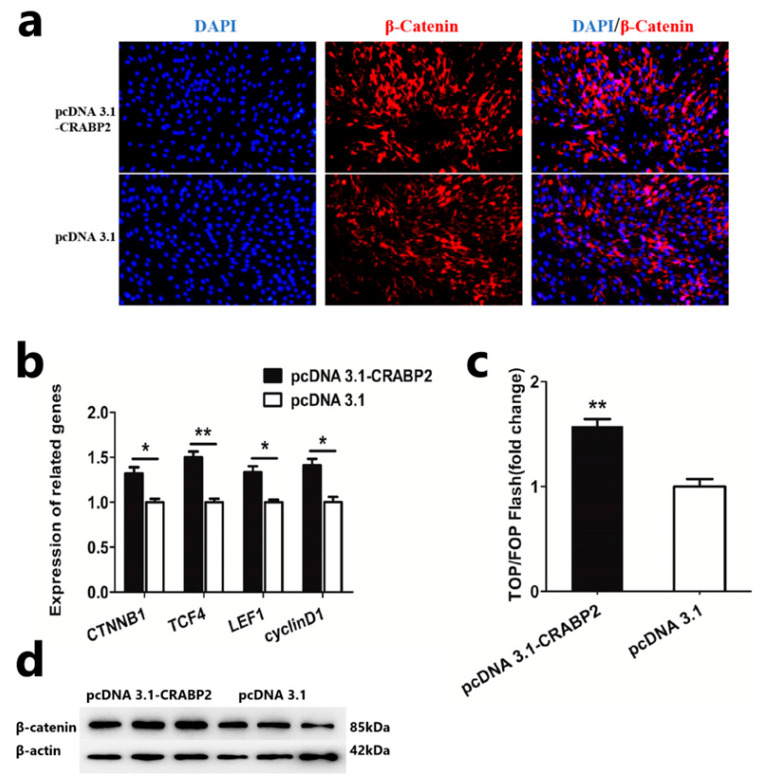
CRABP2 activates the Wnt/β-catenin pathway. (**a**) Immunofluorescence staining of β-catenin after overexpression of CRABP2 in DPCs; the scale is 50 μm. (**b**) The mRNA expression level of Wnt/β-catenin-pathway-related genes after overexpression of CRABP2 in DPCs. (**c**) TOP/FOP-flash assays of Wnt/β-catenin activation after overexpression of CRABP2 in DPCs. (**d**) The protein expression level of β-catenin after overexpression of CRABP2 in DPCs. Data are expressed as means ± SEM (standard error of the mean) (*n* = 3). The unpaired Student’s *t*-test was used for Statistical significance. (* *p* < 0.05; ** *p* < 0.01).

In the proliferation process of DPCs, the Wnt/β-catenin pathway is of great importance. To explore the effect of CRABP2 on the Wnt/β-catenin pathway, we transfected the overexpression vector into DPCs and detected related indicators. Based on the results of immunofluorescence staining, we found that overexpression of CRABP2 could increase the expression level of β-catenin in DPCs (Figure 4a). Then, we detected the expression of Wnt/β-catenin-pathway-related genes (CTNNB1, TCF4, LEF1, and cyclinD1) and β-catenin protein after overexpression of CRABP2 in DPCs. QRT-PCR and Western blot assay showed that overexpression of CRABP2 could increase the mRNA expression level of Wnt/β-catenin-pathway-related genes and the protein level of β-catenin (Figure 4b,d). In addition, the activity of Wnt/β-catenin was significantly increased after overexpression of CRABP2 in DPCs (Figure 4c). All these results suggested that CRABP2 could activate the Wnt/β-catenin pathway of DPCs.

## 4. Discussion

DPCs are mesenchymal cells located in the skin, which are thought to regulate the growth and development of hair follicles by differentiating into different cell types [24,25]. During hair follicle growth, epithelial cell proliferation and new hair follicle downgrowth are first caused by signaling between the placode and the fibroblast condensate [4,26]. Then, the epithelial cell and fibroblast aggregate together to form the mature dermal papilla, and the dermal papilla guides the growth of epithelial cells into the hair shaft and inner root sheath [27]. Some genes have already been reported to participate in the hair growth cycle, such as CRABP1 [15], SOX2 [28], and FGF7/10 [29]. These studies all show that DPCs play an important role in hair follicle growth.

Previous studies showed that CRABP1 is expressed in hair follicles of normal skin (dermal papilla), and CRABP2 is abundantly expressed in hair follicles [14,15,30]. However, the roles of CRABPs in the sheep hair follicle are unclear. In our previous study, we found that CRABP2 was highly expressed in dermal papilla cells compared to other cells in hair follicles [16]. Therefore, we inferred a possible role of CRABP2 in the proliferation of DPCs. Based on the results of qRT-PCR, CCK-8, EdU, and Western blot, we found that overexpression of CRABP2 could increase the proliferation of DPCs in vitro.

The Wnt pathway plays an important role in cell proliferation, differentiation, apoptosis, and anti-apoptosis through intercellular signal transduction, while the canonical Wnt signaling (Wnt/β-catenin pathway) is also the main pathway for DPC proliferation and differentiation [31]. Meanwhile, it is reported that the Wnt/β-catenin pathway can maintain the hair-inducing activity of the dermal papilla and the growth of hair papilla cells [32,33]. Based on these studies, we detected the effect of the Wnt/β-catenin pathway in Hu sheep DPCs. Due to the positive effect of the Wnt/β-catenin pathway on human DPC growth, the inhibition of the Wnt/β-catenin pathway could decrease the proliferation of DPCs, so we assumed a similar effect of the Wnt/β-catenin pathway in Hu sheep DPCs [34]. Therefore, ICG001 was selected and added to DPCs, as it has been proven to inhibit the activity of the Wnt/β-catenin pathway [35]. Then, we found that overexpression of CRABP2 could rescue its activity when the Wnt/β-catenin pathway was inhibited. Moreover, the proliferation of DPCs was resumed to a certain extent after overexpression of CRABP2. All of this revealed that CRABP2 promotes the proliferation of DPCs via the Wnt/β-catenin pathway.

Previous research has shown that the Wnt/β-catenin pathway plays an important role in hair follicle morphogenesis and is related to the expression of CRABP1 and CRABP2 in mice hair follicles [15,36]. However, the effect of CRABP2 on the Wnt/β-catenin pathway in Hu sheep DPCs was still unclear. Therefore, we detected the effect of CRABP2 on the Wnt/β-catenin pathway in DPCs and found that the expression of β-catenin was increased after overexpression of CRABP2 in DPCs. Then, we also found that CRABP2 could activate the Wnt/β-catenin pathway. As a downstream signal molecule in the Wnt pathway, CTNNB1 is related to stem cell proliferation and self-renewal [37]. Another two Wnt downstream factors, TCF4 and LEF1, have also been found to bind to CTNNB1 to co-regulate downstream gene expression [38,39]. Therefore, we selected CTNNB1, TCF4, LEF1, and the Wnt target gene cyclinD1 as the related genes of the Wnt/β-catenin pathway. We also found that CRABP2 could increase the expression of Wnt/β-catenin-pathway-related genes. All of this suggested that CRABP2 could activate the Wnt/β-catenin pathway. This result is also consistent with our previous understanding of the effect of the Wnt/β-Catenin pathway in sheep DPCs [40].

## 5. Conclusions

In our study, we reported that CRABP2 could facilitate the proliferation of DPCs by upregulating the Wnt/β-catenin pathway. The result reveals the positive effect of CRABP2 in the proliferation of Hu sheep dermal papilla cells, broadening the insight into the role of CRABP2 in Hu sheep hair follicles and providing a basis for the functional research of CRABP2 in hair follicles.

## Figures and Tables

**Table 1:** Primers used for qRT-PCR.

Gene	Primer Sequence (5′-3′)	Product Size (bp)	Annealing Temperature (°C)	Accession Number
CRABP2	F: AAAAGTCTCAGCGTCCAGT R: TCAGCATCACATTCACCC	152	60	XM_004002627.5
PCNA	F: CGAGGGCTTCGACACTTAC	97	60	XM_004014340.5
	R: GTCTTCATTGCCAGCACATT			
CDK2	F: AGAAGTGGCTGCATCACAAG R: TCTCAGAATCTCCAGGGAATAG	92	60	NM_001142509.1
CTNNB1	F: GAGGACAAGCCACAGGATTAT	101	60	NM_001308590.1
	R: CCAAGATCAGCGGTCTCATT			
TCF4	F: CACTTTCCCTAGCTCCTTCTTC	136	60	XM_042239261.1
	R: GTAGCTGCTAGACTGTGGAATG			
LEF1	F: CAGGTGGTGTTGGACAGATAA	179	60	XM_042251146.1
	R: ATGAGGGATGCCAGTTGTG			
cyclinD1	F: CCGAGGAGAACAAGCAGATC	91	60	XM_027959928.2
	R: GAGGGTGGGTTGGAAATG			
β-actin	F: GGAATCGTCCGTGACATCAA	107	60	NM_001009784.3
	R: AGCTCGTAGCTCTTCTCCA			

**Table 2:** Primers used for CRABP2 overexpression vector construction.

Primer Name	Primer Sequence (5′-3′)	Product Size (bp)	Annealing Temperature (°C)
OE-CRABP2	F: CTAGCGTTTAAACTTAAGCTTATGCCCAACTTCTCTGGTAACTG	417	62
	R: TGCTGGATATCTGCAGAATTCTTACTCTCGGACATAGACCCTGG		

## Data Availability

All data are contained in the manuscript.
